# Clinical portrait of cochlear implantation in patients with incomplete partition type-III malformation

**DOI:** 10.3389/fped.2025.1682673

**Published:** 2026-01-05

**Authors:** Peng Zhu, Kun Ni, Xiao yan Li, Zheng nong Chen

**Affiliations:** 1 Department of Otorhinolaryngology, Shanghai Jiao Tong University Affiliated Sixth People’s Hospital, School of Medicine, Shanghai Jiao Tong University, Shanghai, China; 2 Department of Otorhinolaryngology, Shanghai Children’s Hospital, School of Medicine, Shanghai Jiao Tong University, Shanghai, China; 3 Department of Otorhinolaryngology, Shanghai Children’s Hospital, School of Medicine, Shanghai Jiao Tong University, Shanghai, China; 4 Department of Otorhinolaryngology, Shanghai Jiao Tong University Affiliated Sixth People’s Hospital, School of Medicine, Shanghai Jiao Tong University Shanghai, Shanghai, China

**Keywords:** incomplete partition type III (IP-III), inner ear malformation, cochlear implantation, intraoperative imaging, enlarged internal auditory canal

## Abstract

**Objective:**

To investigate the clinical characteristics and postoperative speech development in children with incomplete partition type III (IPIII) cochlear malformation.

**Methods:**

A retrospective analysis was conducted on 6 patients (12 ears) diagnosed with IPIII cochlear malformation, all patients underwent preoperative high-resolution CT (HRCT), middle ear and cranial MRI, and audiological evaluations. Intraoperative cochlear imaging was performed to confirm electrode placement, there was also 1 types of comparison: IP III patients with matched CI patients without inner ear malformations, questionnaires were used to evaluate auditory and speech perception

**Results:**

All 6 patients were male, with 3 undergoing bilateral cochlear implantation and 3 undergoing unilateral implantation (1 left, 2 right). The surgical age ranged from 8 months to 12 years, with a median age of 17 months. HRCT findings in all 12 ears (100%) showed a grossly normal cochlear shape with bony cochlear partitions present but an absent modiolus. Ten ears (10/12; 83.3%) exhibited enlarged internal auditory canals (6.13 ± 0.395 mm), 9 ears (9/12; 75%) had profound sensorineural hearing loss, 4 ears (4/12; 33.3%) showed significant vestibular dilation with a cystic appearance, and 4 ears (4/12; 33.3%) had enlarged vestibular aqueducts. All patients had normal auditory nerve development (100%). intraoperative neural response telemetry (NRT) responses were successfully elicited in all cases, and all patients experienced gusher phenomenon (6/6; 100%). Intraoperative cochlear imaging preliminarily confirmed electrode placement. Follow-up ranged from 3 months to 1 year, with preoperative CAP scores 0.83 ± 0.41 and postoperative CAP scores 6 ± 1.55, p<0.05. In Speech Intelligibility Rating (SIR) questionnaires, CI patients without inner ear malformations outperformed IP III patients, while there was no significant difference in other questionnaires.

**Conclusion:**

IPIII cochlear malformation is more common in male patients and is often associated with profound to severe sensorineural hearing loss. Cochlear implantation for IP-III malformation leads to significant auditory-speech improvement in early stage and can result in varying degrees of oral competence.

## Introduction

Cochlear Incomplete Partition Type III (IP-III) is a rare inner ear malformation, accounting for 2.0%–4.8% of all inner ear anomalies (1, 2). It was first classified as a subtype of incomplete partition malformations by Sennaroglu et al. in 2002 (3). IP-III deafness is also known as X-linked hereditary hearing loss and is associated with POU3F4 gene mutations (4). Clinically, it often presents as bilateral severe-to-profound sensorineural hearing loss (SNHL). Currently, cochlear implantation (CI) is the primary treatment modality. However, intraoperative complications such as cerebrospinal fluid (CSF) gusher and electrode misplacement into the internal auditory canal (IAC) are common (5). Preoperative comprehensive evaluation of clinical features through imaging and audiological assessments is essential to develop an appropriate treatment strategy (6).

This study included 6 pediatric patients with incomplete partition type III (IP-III) inner ear malformation who underwent cochlear implantation (CI). We analyzed their imaging and audiological characteristics, summarized the common intraoperative complications, and evaluated postoperative speech rehabilitation outcomes. The aim was to enhance the understanding and treatment of this rare inner ear anomaly.

## Materials and methods

We conducted a retrospective analysis of 6 pediatric patients (12 ears) diagnosed with incomplete partition type III (IP-III) cochlear malformation who underwent cochlear implantation at Shanghai Children's Hospital from January 2023 to January 2025 (Table 1). All patients underwent high-resolution CT (HRCT GB LightSpeed 64-slice CT, the slice interval was set to 0.625 mm), Magnetic Resonance Imaging(MRI, Philips Healthcare Ingenia 3.0 T), and audiological examinations before surgery. the internal auditory canal (ICA) is deemed enlarged if it measures greater than 5 mm at its midpoint (7), intraoperatively, a plain x-ray of cochlear view was performed to confirm the position of the cochlear electrode, and postoperative middle ear CT(Axial) was conducted to reconfirm the electrode placement. The CAP (Categories of Auditory Performance) scale was used to assess the speech development of the children both before and after the procedure. Patients who underwent cochlear implantation without obvious inner ear malformations during the same period were selected as the control group, 3 of these patients underwent bilateral cochlear implantation and 3 undergoing unilateral implantation, the surgical age ranged from 7 months to 12 years, with a median age of 15 months. Questionnaires were used to evaluate auditory and speech perception one year later after the surgery (8), including the Infant-Toddler Meaningful Auditory Integration Scale, Meaningful Auditory Integration Scale (IT-MAIS/MAIS), Categories of Auditory Performance (CAP), Speech Intelligibility Rating (SIR), Meaningful Use of Speech Scale (MUSS),all these questionnaires was Chinese version, and the scoring was blinded by the same one doctor.

**Table 1:** Clinical characteristics and preoperative audiology test results of individual patients.

Case number	Sex	Age at CI, mo	Pou3f4 mutation	Implanted ear	Cochlear implant types	CAP before surgery
1	M	48	Not done	Left	CI 622	1
2	M	144	+	Bilateral	SYNCHRONYMi1200	1
3	M	24	Not done	Right	SYNCHRONY Mi1200	0
4	M	8	Not done	Bilateral	SYNCHRONY Mi1200	1
5	M	10	Not done	Bilateral	CI 522	1
6	M	10	+	Right	CI 622	1

### Statistical analysis

Data analysis was performed using SPSS 26.0 statistical software(International Business Machines Corporation). The Kolmogorov–Smirnov test was used for normality testing. Since age did not follow a normal distribution, it was expressed as the median. Categorical data were presented as frequencies and percentages. When comparing the preoperative and postoperative CAP (Categories of Auditory Performance) scores, the Mann—Whitney U test was used. *T*-test was used to determine the difference between the IP III patients and without inner ear malformations, *p* < 0.05 was considered to indicate a statistically significant difference.

## Results

**Figure 1:**
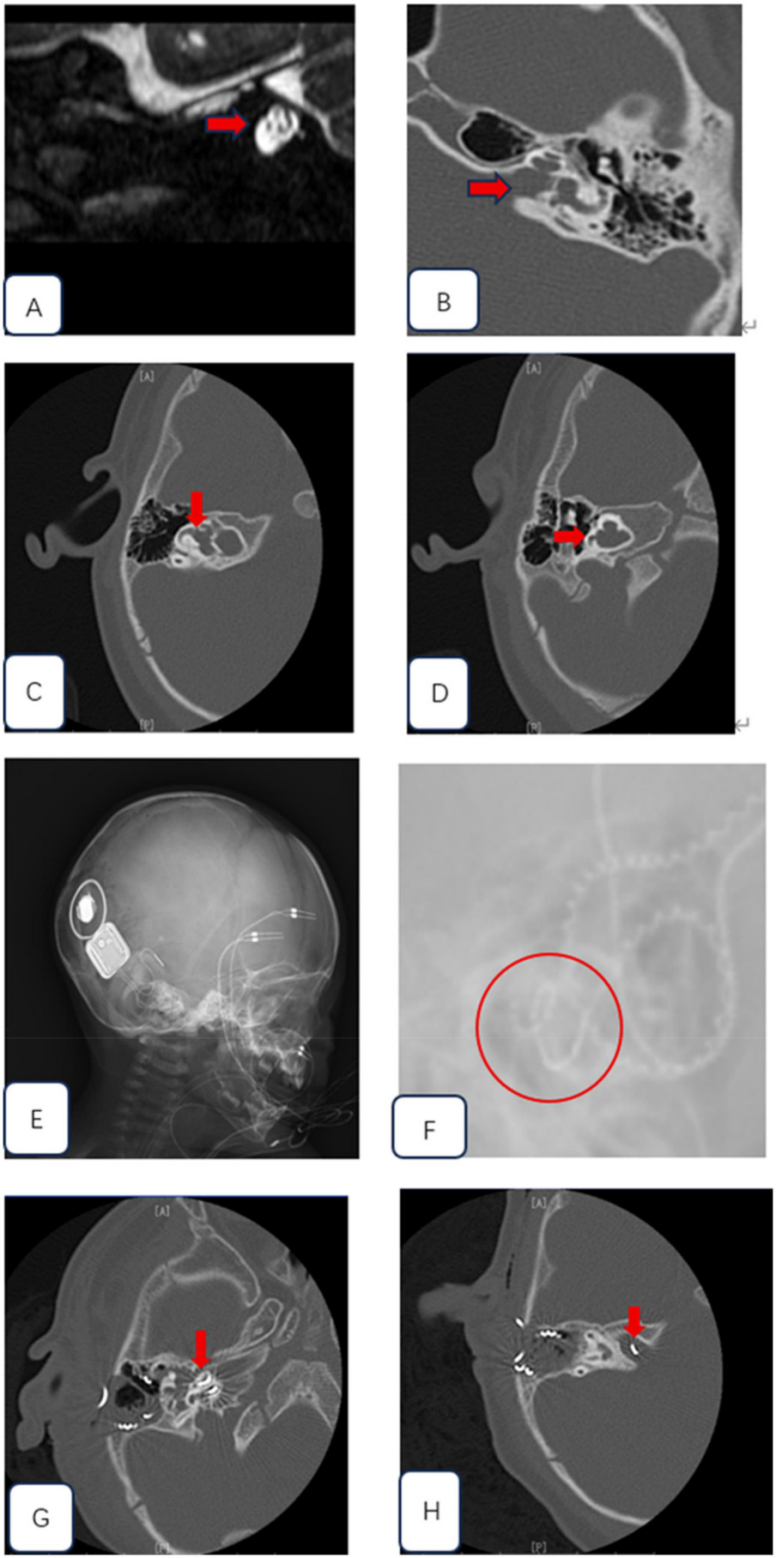
**(A)** axial, T2-weighted MR imaging of the internal auditory canal (ICA) suggest that all children had normally developed auditory nerves **(B)** widened internal auditory canal (IAC) **(C)** dilation of the vestibule with a cystic appearance **(D)** the cochlea was generally normal, the bony cochlear partition was present, and the modiolus was absent **(E)** cochlear imaging was used to confirm the position of the cochlear electrodes **(F)** intraoperative x-ray showed optimal placement of the cochlear electrode **(G)** postoperative CT scan confirmed the optimal position of the cochlear electrode H postoperative CT scan suggests misplacement of the cochlear implant electrode into the internal auditory canal.

**Figure 2:**
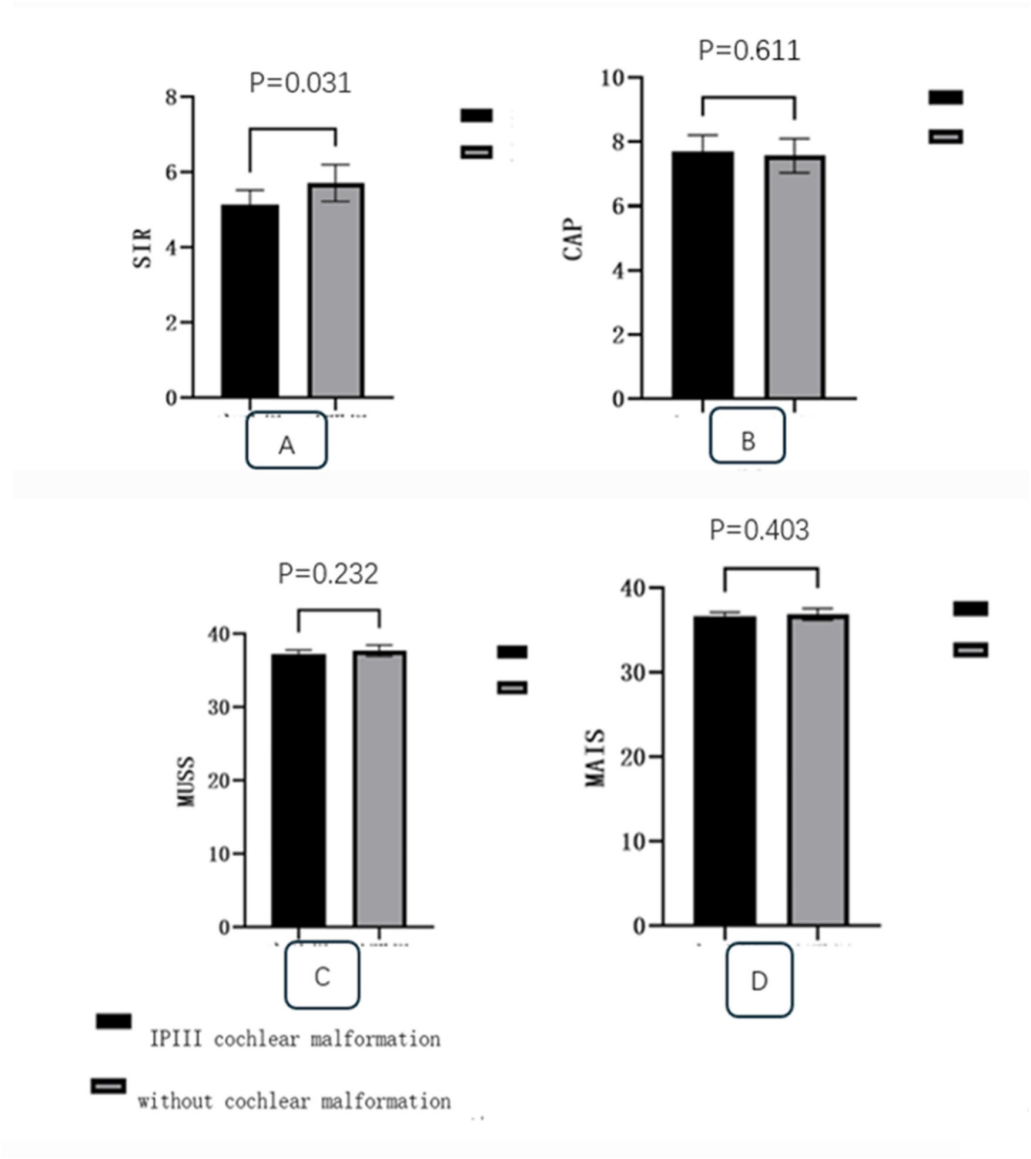
In speech intelligibility rating (SIR) questionnaires, CI patients without inner ear malformations outperformed IP III patients **(A)**, while there was no significant difference in other there questionnaires **(B–D)**.

All six patients were male, with three undergoing bilateral cochlear implantation and three undergoing unilateral cochlear implantation (1 left, 2 right) (Table 2). The surgical age ranged from 8 months to 12 years, with a median age of 17 months. HRCT scans of all 12 ears (100%) in the six patients showed that the external shape of the cochlea was generally normal, the bony cochlear partition was present, and the modiolus was absent (figur1-D). ten ears (10/12; 83.3%) exhibited widening of the internal auditory canal (6.13 ± 0.395 mm) (Figure 1B), nine ears (9/12; 75%) had profound sensorineural hearing loss, four ears (4/12; 33.3%) had significant dilation of the vestibule with a cystic appearance (Figure 1C), and four ears (4/12; 33.3%) had an enlarged vestibular aqueduct. All children had normally developed auditory nerves (100%) (Figure 1A). Due to financial reasons, many parents decline genetic testing before surgery. Finally two patients underwent genetic testing, and both were found to have mutations in the POU3F4 gene. Surgery was performed via the round window (Table 3). The electrode array was be inserted completely in all these patients, and intraoperative NRT responses were successfully elicited in all cases. All patients experienced perilymph gushers during surgery (6/6; 100%), and temporalis fascia autograft was used to seal the opening at the the round window site. cochlear imaging was performed intraoperatively to preliminarily confirm the position of the cochlear electrodes (Figures 1E,F). Postoperative middle ear CT scans showed that in five patients (eight ears; 88.9%; three bilateral, two unilateral), the electrode positions were satisfactory (Figure 1G), while in one ear (1/9; 11.1%), the electrode protruded into the internal auditory canal (Figure 1H), and a second cochlear implantation surgery was required to correct the position of electrode, no complications such as cerebrospinal fluid otorrhea occurred in any of the patients postoperatively. the follow-up period ranged from 3 months to 1 year. Preoperative CAP values0.83 ± 0.41 and postoperative CPA values 6 ± 1.55are provided, *p* < 0.05, in Speech Intelligibility Rating (SIR) questionnaires (4.143 ± 0.378 VS 4.714 ± 0.488 *p* < 0.05), CI patients without inner ear malformations outperformed IP III patients, while there was no significant difference in other there questionnaires ( CAP 7.714 ± 0.488 VS 7.571 ± 0.522, MUSS 37.286 ± 0.488 VS 37.714 ± 0.756, MAIS 36.571 ± 0.535 VS 36.857 ± 0.69 *P* > 0.05) (Figure 2).

**Table 2:** Radiologic findings and associated anomalies of individual patients.

Case number	abr	oae	assr	Cochlear anomaly	Enlargement of Scc	CN anomaly	Enlargement of ICA	Enlargement of vestibular aqueduct
1L	90	NR	100	IP-III	+	none	–	–
1R	100	NR	100	IP-III	+	none	+	–
2L	100	NR	100	IP-III	+	none	+	–
2R	100	NR	100	IP-III	+	none	+	–
3L	80	NR	80	IP-III	+	none	+	+
3R	100	NR	100	IP-III	+	none	+	+
4L	100	NR	100	IP-III	+	none	+	+
4R	100	NR	100	IP-III	+	none	–	+
5L	100	NR	100	IP-III	+	none	+	–
5R	85	NR	90	IP-III	+	none	+	–
6L	70	NR	80	IP-III	+	none	+	–
6R	100	NR	100	IP-III	+	none	+	–

**Table 3:** Intraoperative findings and surgical outcomes of individual patients.

Case number	Approach	route	ECAP	gusher	Cerebrospinal fluid otorrhea	Facial paralysis	The working status of the CI	CAP
1L	Facial recess	RW	Partial response	＋	–	–	Functional	8
2L	Facial recess	RW	Partial response	＋	–	–	Functional	7
2R	Facial recess	RW	Partial response	＋	–	–	Functional	
3R	Facial recess	RW	Partial response	＋	–	–	Functional	5
4L	Facial recess	RW	Partial response	＋	–	–	Functional	5
4R	Facial recess	RW	Partial response	＋	–	–	Functional	
5L	Facial recess	RW	Partial response	＋	–	–	Functional	7
5R	Facial recess	RW	Partial response	＋	–	–	Functional	
6R	Facial recess	RW	Partial response	+	–	–	Functional	4

## Discussion

Human inner ear development is typically completed around 28 weeks of gestation. Congenital inner ear malformations can occur at various stages prior to the completion of embryonic development (9, 10), genetic factors, viral infections, and other adverse physicochemical factors may all contribute to these malformations. The incidence of inner ear malformations in the general population is approximately 1/2,000–1/6,000 (3). Such malformations are a common cause of congenital sensorineural hearing loss (SNHL), with 20%–30% of children with severe-to-profound congenital SNHL showing abnormal inner ear structures on imaging studies (11). For children with severe-to-profound SNHL, cochlear implantation (CI) is currently the most effective treatment. However, in cases of severe congenital inner ear malformations, CI surgery may carry significant risks, including uncertain outcomes, intraoperative complications, and postoperative adverse events.

Inner ear malformation of type III incomplete partition (IP—III) is considered to be associated with POU3F4 on the X chromosome (12). It was first reported by Nance et al. (13) in 1971 in male patients with X—linked deafness. All the patients were male and had severe to profound sensorineural hearing loss. Female carriers had milder symptoms. However, subsequent studies have also found female patients with typical clinical symptoms and imaging manifestations. Phelps et al. (14) first described its high—resolution computed tomography (HRCT) manifestations, but it was not included in the classification of incomplete partition cochlear malformations until 2006 (15).

In this study, all affected ears showed abnormal morphology of the cochlea and internal auditory canal. The external shape of the cochlea was roughly normal, the bony partition of the cochlea existed, but the modiolus was absent. the bottom of the internal auditory canal was dilated, there was no partition between the internal auditory canal and the cochlea, and it was directly connected to the basal turn, presenting a “gourd—like” appearance. Meanwhile, IP—III cochlear malformation was also accompanied by structural abnormalities of the vestibule and vestibular aqueduct to varying degrees. however, the development of the cochlear nerve was normal, which was consistent with previous research results.

Cochlear implantation (CI) is the most effective treatment for severe to profound sensorineural hearing loss. however, IP—III inner ear malformation was previously considered a relative contraindication for CI (16). with the improvement of CI surgical techniques and the development of cochlear implant devices, CI is currently regarded as an effective method to improve the hearing of children with IP—III inner ear malformation.

Due to the absence of the modiolus and the partition at the bottom of the cochlea near the bottom of the internal auditory canal in IP—III malformation, the difficulty of electrode implantation during surgery and the risk of complications increase (17). During electrode implantation, the electrode may accidentally enter the internal auditory canal, resulting in implantation failure. It is crucial to refer to preoperative imaging examination results to select an appropriate implantation path and electrode. During the operation, when implanting the electrode, its tip should avoid being directly facing the internal auditory canal. Combining intraoperative neural response telemetry (NRT) examination and cochlear anteroposterior and lateral X—rays can initially clarify the position of the implanted cochlear electrode, reducing the risk of re—revision surgery. After the operation, cochlear CT examination is required to re—confirm the position of the implanted electrode (7).

In this study, among 9 ears of 6 patients, only 1 ear in 1 patient had the electrode accidentally entering the internal auditory canal, while the positions of the electrodes in the other 8 ears of 5 patients were appropriate. during cochlear implantation surgery for IP—III malformation, gusher is very likely to occur. Phelps et al. (16) believed that it was because the subarachnoid space in the internal auditory canal was connected to the perilymph in the cochlea. During surgery, cerebrospinal fluid directly entered the cochlea through the bony defect, resulting in a rapid increase in pressure in the cochlea and causing gusher. Intraoperative gusher may lead to unsuccessful electrode implantation. however, some scholars believe that electrode implantation during gusher can prevent the electrode from entering the internal auditory canal (7). In this group of 9 ears in 6 patients, gusher occurred during all implantation surgeries. Temporalis fascia was used to seal the opening at the cochleostomy site, and no complications such as cerebrospinal fluid rhinorrhea occurred after the operation.

Patients with IP—III malformation have scattered residual spiral ganglion cells in the cochlea, which may also lead to suboptimal postoperative speech rehabilitation outcomes. Some scholars believe that using short electrodes or full—ring electrodes that only encircle the cochlea once, combined with intraoperative CT scanning or robotic technology, can effectively stimulate the spiral ganglion cells and avoid accidental insertion of the electrode into the internal auditory canal, thereby achieving better hearing compensation after the operation (7).

For patients with severe to profound sensorineural hearing loss, preoperative thorough reading of temporal bone CT scans, in combination with inner ear MRI, is necessary for making a clinical diagnosis. For patients with severe inner ear malformations, such as those with IP—III malformation, cochlear implantation is no longer a contraindication. Before the operation, the surgical plan should be carefully discussed, and patients and their families should be thoroughly informed about possible complications and prognoses. Meanwhile, the surgeon is required to possess superb microsurgical skills, rich experience in cochlear implantation for patients with inner ear malformations, in—depth knowledge of temporal bone anatomy, and high—quality surgical equipment to achieve satisfactory clinical treatment outcomes.

### Limitations

This study has limitations, first, the overall sample size was relative small, the follow-up time was short, and the genetic testing performed in only 2 patients, in the future, a standardized prospective continuous follow-up in these patients with a bigger sample size should be established.

## Conclusion

IP—III cochlear malformation is clinically rare and more commonly seen in male patients. It is often associated with vestibular and internal auditory canal malformations. Intraoperative gusher and accidental electrode insertion into the internal auditory canal are likely to occur. Cochlear implantation (CI) is an effective method to improve the postoperative hearing of children with IP—III inner ear malformation.

## Data Availability

The original contributions presented in the study are included in the article/Supplementary Material, further inquiries can be directed to the corresponding authors.
